# Seasonality of *Nosema ceranae* Infections and Their Relationship with Honey Bee Populations, Food Stores, and Survivorship in a North American Region

**DOI:** 10.3390/vetsci7030131

**Published:** 2020-09-08

**Authors:** Berna Emsen, Alvaro De la Mora, Brian Lacey, Les Eccles, Paul G. Kelly, Carlos A. Medina-Flores, Tatiana Petukhova, Nuria Morfin, Ernesto Guzman-Novoa

**Affiliations:** 1 School of Environmental Sciences, University of Guelph, 50 Stone Road East, Guelph, ON N1G 2W1, Canada; bernaemsen@gmail.com (B.E.); alvarodlmr@gmail.com (A.D.l.M.); pgkelly@uoguelph.ca (P.G.K.); nmorfinr@uoguelph.ca (N.M.); 2 Department of Animal Science, Agricultural Faculty, Ataturk University, Erzurum 25240, Turkey; 3 Ontario Beekeepers’ Association Technology Transfer Program, 185, 5420 Hwy 6 N, Orchard Park Office, Guelph, ON N1H 6J2, Canada; brianwlacey@gmail.com (B.L.); les.eccles@ontariobee.com (L.E.); 4 Unit of Veterinary Medicine and Animal Science, University of Zacatecas, Carr. Panamericana Km 35.5, El Cordovel 98500, Zac., Mexico; carlosmedina@uaz.edu.mx; 5 Department of Population Medicine, University of Guelph, 50 Stone Road East, Guelph, ON N1G 2W1, Canada; tpetukho@uoguelph.ca

**Keywords:** *Nosema ceranae*, viability, prevalence, infection intensity, seasonality, bee longevity, bee population, honey stores, CCD

## Abstract

*Nosema ceranae* is an emerging pathogen of the western honey bee (*Apis mellifera* L.), and thus its seasonality and impact on bee colonies is not sufficiently documented for North America. This study was conducted to determine the infection intensity, prevalence, and viability of *N. ceranae* in >200 honey bee colonies during spring, summer, and fall, in a North American region. We also determined the relationship of *N. ceranae* infections with colony populations, food stores, bee survivorship, and overwinter colony mortality. The highest rates of *N. ceranae* infection, prevalence, and spore viability were found in the spring and summer, while the lowest were recorded in the fall. *N. ceranae* spore viability was significantly correlated with its prevalence and infection intensity in bees. Threshold to high levels of *N. ceranae* infections (>1,000,000 spores/bee) were significantly associated with reduced bee populations and food stores in colonies. Furthermore, worker bee survivorship was significantly reduced by *N. ceranae* infections, although no association between *N. ceranae* and winter colony mortality was found. It is concluded that *N. ceranae* infections are highest in spring and summer and may be detrimental to honey bee populations and colony productivity. Our results support the notion that treatment is justified when infections of *N. ceranae* exceed 1,000,000 spores/bee.

## 1. Introduction

The microsporidian parasite *Nosema ceranae* is an emerging pathogen of the western honey bee (*Apis mellifera* L.), and thus many aspects of its biology, pathology, and impact on colony conditions are unknown or not sufficiently documented. It is believed that the original host of *N. ceranae* is the Asiatic bee, *Apis cerana* [1], and it recently jumped to *A. mellifera* [2,3], which has been frequently infected by another microsporidian, *N. apis*, for more than a century [4]. *N. ceranae* and *N. apis* cause nosema disease in honey bees by parasitizing their midgut epithelial cells and impairing the digestion and absorption of nutrients in the infected individuals [5,6].

Spores of *Nosema* spp. can be observed and quantified by microscopy, but to identify the *Nosema* species observed, it is necessary to use molecular methods of detection. The most common molecular methods of *Nosema* spp. detection and quantification are those that use primers for the *16s rRNA* gene [1], which has multiple copies in the *Nosema* genome. More reliable methods of detection and quantification based on the single-copy gene *Hsp70* have been recently developed [7].

Nowaday, *N. ceranae* is distributed worldwide and is the microsporidian that is most frequently found in honey bee colonies, even on islands distant from mainland countries [6,8,9]. However, information on seasonal variation of *N. ceranae* infections, prevalence, and spore viability, as well as about its impact on colony conditions and bee survivorship, is particularly scarce for North America. Surveys in Europe have shown that the prevalence and intensity of *N. ceranae* infections are not constant throughout the year, and fluctuate between seasons and geographical regions [10]. However, the prevalence of *N. ceranae* in honey bee colonies does not show seasonality in Spain [11,12]. In the USA, a 12-month study showed that the intensity of *N. ceranae* infections in honey bee colonies vary seasonally in a similar way *N. apis* infections vary, with the highest spore counts of the parasite observed in the spring, and the lowest in the fall and winter [13]. Likewise, in Western Canada, Ibrahim et al. [14] reported that *N. ceranae* and *N. apis* showed a similar pattern of infection as that described for honey bee colonies in the USA [13]. However, in North America there is no information on whether the viability of *N. ceranae* spores shows a seasonal pattern and if this pattern is related to the intensity and prevalence of infections within colonies. It is also unknown how these patterns affect disease morbidity and colony conditions. This information is necessary for designing control strategies against the microsporidian.

Most of what is known about the epidemiology of nosema disease has been derived from studies with *N. apis*, and it is assumed that it must be similar for *N. ceranae* infections. However, this information needs to be corroborated with studies conducted with *N. ceranae* in different geographical locations. Therefore, in addition to determining seasonal changes in the intensity of *N. ceranae* infections, it is important to determine if these differences are related to the prevalence of infected bees within a colony, as well as to the viability of the spores at different times of the year. The viability of *Nosema* spp. spores influences their ability to infect bees and to multiply [15]. Therefore, determining the viability of the parasite’s spores provide an indication of its ability to cause disease. This information will help to better understand the seasonality of infections in different locations and to determine the optimal treatment times for the control of nosema disease caused by *N. ceranae* in honey bee colonies.

The pathogenicity and virulence of *N. ceranae* to honey bees has been analyzed in numerous studies. In the majority of them, *N. ceranae* has been found to significantly shorten the lifespan of bees [16,17,18,19,20], although some have not found such effect (see, e.g., Huang et al. [21]). However, most of these studies have been conducted under laboratory conditions using caged bees, which are useful to study certain aspects of nosema disease, but cage experiments are probably not very suitable to study the effects of the parasite on bee longevity because they are conducted under artificial conditions. Studies under the natural environment of colonies within hives are thus required to confirm the impact of *N. ceranae* on bee longevity.

At the hive level, several studies conducted in Spain have related *N. ceranae* infections to colony collapse [22,23,24]. Similarly, another study suggested that *N. ceranae* was one of the factors that predicted seasonal colony mortality in Switzerland [25]. However, in other European countries, no clear causal link between the microsporidian and colony failure has been evidenced [10,26,27,28]. Likewise, the studies conducted in Canada, continental USA, and Hawaii did not find a relationship between *N. ceranae* infection and winter colony losses [29,30,31,32]. Nevertheless, the levels of *N. ceranae* infections were higher than those of *N. apis* infections in two Canadian provinces [33]. Similarly, *N. ceranae* has not been associated to colony collapse in Mexico and South America [34,35,36]. Apparently, data on the virulence and impact of *N. ceranae* at the colony level are contradictory and focused on the collapse of colonies rather than on the effect of the parasite on colony strength and food stores. The conflicting findings between studies conducted in different regions could be due to differences in virulence of *N. ceranae* isolates, differences in susceptibility of bee strains, or differences in other unknown factors [37].

Because seasonal and regional differences may be important for the pathogenicity and virulence of *N. ceranae*, additional studies on the epidemiology and impact of the microsporidian need to be conducted at the colony level in different regions of the world. Therefore, this study was conducted to determine the seasonal patterns of *N. ceranae* infection, prevalence, and viability in honey bees from commercial colonies in Ontario Canada. We also studied the relationship of *N. ceranae* infections with colony populations, food stores, and overwinter survival, as well as how the parasite affects the lifespan of worker bees in colonies.

## 2. Materials and Methods

### 2.1. Screening and Selection of Experimental Colonies

Approximately 100 worker bees per sample were initially collected with a bee vacuum [38] from the hive entrance of more than 300 colonies of commercial beekeepers from Southern Ontario, Canada in early spring. These colonies had not been treated against nosema disease during the last 12 months. The samples were stored at −20 °C and later microscopically analyzed for the presence of *Nosema* spp. spores [39]. Randomly chosen subsamples of 10 bees from each of the positive samples were subsequently subjected to PCR tests to detect *N. ceranae* infections [40]. Colonies infected with *N. ceranae* only (*n =* 233) were selected and sampled during different seasons (mid-spring, mid-summer, and mid-fall) to determine infection intensity of *N. ceranae* in all of them. Ten of the selected colonies were randomly chosen to provide subsamples of 30 bees/colony for assessments of *N. ceranae* spore prevalence and viability during each season. The selected 233 colonies in this study were managed similarly and treated against *Varroa destructor* mites (Apivar^®^, Veto-Pharma) and preventatively against foulbrood diseases (Oxytet-25^®^, Medivet) in the fall, according to the instructions of the manufacturers. No visual clinical signs of brood diseases were detected in the colonies during the course of the study. Treatments against nosema disease were not applied.

### 2.2. Infection Intensity

The intensity of *N. ceranae* infections was determined in samples of 60 forager bees as recommended by the OIE [41]. The bees were collected from the selected colonies (*n =* 233, 227, and 196, for spring, summer, and fall, respectively) as above described. The microscopic assessment of *N. ceranae* infection intensity was done using a phase contrast microscope (Olympus BX41; Olympus, Markham, ON, Canada) and a hemocytometer to obtain *Nosema* spores counts, which were used for calculations and recorded as mean number of spores per bee [39]. Spore counts are a reliable method of determining the intensity of *N. ceranae* infections because they correlate with PCR quantifications [40,42], another way of determining *Nosema* infection levels. Previously, we had demonstrated a high and significant linear relationship (R^2^ = 0.95) between *N. ceranae* copies quantified by PCR, and number of spores from samples of bees with different infection levels of *N. ceranae* [33].

### 2.3. Spore Viability

To determine the viability of *N. ceranae* spores, three replicates of fresh spores were obtained from 30 bees of each of the 10 positive colonies that were chosen for these assessments. The spores were extracted and purified as per McGowan et al. [43]. After extraction, 10 µL of the spore suspension was used to count spores to determine their concentration in the suspension [39]. The *N. ceranae* spore concentrations were adjusted to obtain a final concentration of 1 × 10^5^ spores/µL and kept in 1.5 mL microcentrifuge tubes at 5 °C until needed. Spores were stained with fluorescent 4′,6-diamino-2-phenylindole (DAPI; Boheringer Manheim Biochemicals, Indianapolis, USA [44]) and propidium iodide (PI; Boheringer Manheim Biochemicals, Indianapolis, USA [45]) dyes as described by McGowan et al. [43]. Briefly, 200 µL of spore suspension was placed in a microcentrifuge tube and incubated simultaneously with 20 µL of each of the dye solutions (1 mg/mL in ddH_2_O) at room temperature for 20 min. After incubation, the spores were rinsed twice by centrifugation at 800 rpm for 6 min in 100 µL of ddH_2_O. The final pellet was suspended in 100 µL of ddH_2_O. A smear was prepared on a microscope slide with 5 µL of the spore suspension and 5 µL of ProLong^®^ Gold reagent (Antifade, Invirtrogen, Burlington, ON, Canada) to protect the fluorescent dyes from fading. Subsequently, the smear was allowed to partially dry for 5 to 10 min, and a cover slip was placed on the smear. The slides were kept in the dark overnight and were sealed the next day using clear nail varnish.

Stained spores were observed at 400× magnification under a fluorescent microscope (Leica DM5500B, Leica Microsystems, Wetzlar, Germany). Between 25 and 30 different fields, containing approximately 30–40 spores per field, were observed using the bright field and the fluorescent light filters for DAPI and PI, and were photographed with a digital camera mounted on the microscope (Hamamatsu Photonics K.K., Hamamatsu, Japan). The photographs were analyzed with an imaging program (Volocity^®^ version 5.1, Improvision, Coventry, UK) to compare the total number of spores between the bright field and the fluorescent filters. The bright light field provided the total number of spores, which are seen as refracting oval corpuscles. The DAPI fluorescent filter provided the number of total spores, which are blue-stained, indicating that they are indeed spores because their walls have been stained. The PI fluorescent filter provided the number of nonviable spores, which are observed red, indicating that the cell wall of the spore is compromised, allowing the dye to penetrate and stain its DNA. The percentage of viable spores was determined by subtracting the number of red spores counted under the PI fluorescent filter (nonviable spores), from the total number of spores counted under the bright field (which is roughly equal to the number of spores counted with the DAPI filter), and dividing this figure by the total number of spores. The resulting figure was then, multiplied by 100.

### 2.4. Nosema ceranae Prevalence in Bees (Percentage of Infected Bees)

The presence or absence of *N. ceranae* spores was determined individually in 30 bees per sample using microscopy as previously described. The percentage of infected bees was calculated by dividing the number of positive bees (having observable *Nosema* spores) by the total number of bees in the sample (30) and the result multiplied by 100.

### 2.5. Colony Evaluations and Relationship with Nosema ceranae Infections

Each of the selected colonies was assessed in the following manner. The colonies were opened and a frame by frame visual estimate to the 0.25 fraction level of the number of frames covered with bees or having brood, stored pollen, or stored honey was recorded [46]. Additionally, to detect possible relationships between different levels of *N. ceranae* infections and colony conditions, the colonies were arbitrarily classified as having low (<1,000,000 spores/bee), threshold (>1,000,000 <2,000,000 spores/bee), and high (>2,000,000 spores/bee) *N. ceranae* infection levels using the data from summer evaluations (the most productive season for the bees). This classification was partially based on estimations of the severity of *N. apis* infections by studies establishing the treatment threshold in 1,000,000 spores/bee [47,48,49], as this threshold has not been established for *N. ceranae* yet. Then, comparisons between the three categories of colonies were done for bee populations, brood amount, pollen stores and honey stores. Additionally, the population growth of colonies between spring and summer was determined by subtracting the number of frames covered by bees or containing brood of the summer assessments from that of the spring assessments. Data on colony growth rates were correlated with *N. ceranae* infections and colony conditions. In the following spring, colony evaluations were conducted as above for overwintered colonies, and the percentage of surviving colonies was determined.

### 2.6. Effect of Nosema ceranae Infections on Bee Survivorship in Colonies

The size of bee populations and colony productivity depend highly on how long bees live [50,51]. Therefore, it seemed important to determine how *N. ceranae* infections affect the survivorship of bees in the natural environment of a colony, rather than in cages within a laboratory, as most previous studies on bee longevity have been conducted. However, determining bee survivorship of marked bees in a colony is challenging. Therefore, three observation hives were used to facilitate daily censuses of the experimental bees. Each observation hive (47.0 × 4.1 × 96.5 cm) was installed in a laboratory and was connected to the exterior of the building to allow the bees to forage. Four newly drawn combs with ~3000 worker bees of variable ages and a queen unrelated to the experimental bees were installed into each hive. Newly emerged bees that were inoculated or that were not inoculated with *N. ceranae* spores were introduced to the observation hives. To obtain newly emerged bees, brood combs from three colonies were placed overnight in wire emerging cages inside an incubator (33 ℃, 70% RH). The next morning, the newly emerged bees were transferred to a plastic container and *Nosema* spore diagnosis was performed as before in a subsample of them to ensure that the bees were *Nosema* spore free. Emerged bees were initially starved for 2 h and then force-fed 5 µL of a 50% sucrose syrup that contained *N. ceranae* spores at a concentration of 1 × 10^4^ spores/µL, using a micropipette (Eppendorf, Mississauga, ON, CA). The suspension of spores used for survivorship experiments was confirmed to only contain *N. ceranae* spores [40]. Control bees only received 5 µL of the syrup without spores. The experimental bees were identified by marking them with enamel paint of different colors (according to treatment) on their thoraces.

Approximately 1000 marked bees—500 inoculated and 500 non-inoculated with *N. ceranae* spores—were introduced into each observation hive. By co-fostering the bees of both treatments in a common hive, environmental influences were minimized [52] to more accurately measure the effect of *N. ceranae* infection on bee survivorship. After introduction, bee acceptance was determined by visually censing the marked bees as per Unger and Guzman-Novoa [53] 24 h after introducing the bees to the observation hives. Subsequently, censuses were performed every day during early mornings when bees were not foraging, from day two after introduction, until day 35. Bee survivorship was estimated by calculating the proportion of bees of each treatment that were present in each hive every day over the initial number of accepted bees. At the end of the study, the surviving marked bees of each treatment in the observation hives were collected and analyzed for *N*. *ceranae* spores as a pooled sample, to confirm that infections developed normally in the inoculated bees.

### 2.7. Statistical Analyses

Percentage data (viability and prevalence) were arcsine-square root transformed, whereas the data on infection intensity were log transformed to normalize them. Data on colony populations and food stores were square root transformed. Transformed data were subjected to analysis of variance (ANOVA) and the means were compared with Tukey tests at a *p* < 0.05 when significance was detected. Additionally, data on infection intensity, prevalence, viability, colony conditions and bee population growth were analyzed with Spearman rank correlations. Data on bee survivorship were analyzed with the Kaplan–Meier method, comparing estimated survival functions. Survival curves were compared with a long-rank test. Differences in colony mortality after the winter were tested with χ^2^ tests. All statistical analyzes were performed using the R statistical program [54].

## 3. Results

### 3.1. Intensity, Prevalence, and Viability of Nosema ceranae Infections

**Figure 1:**
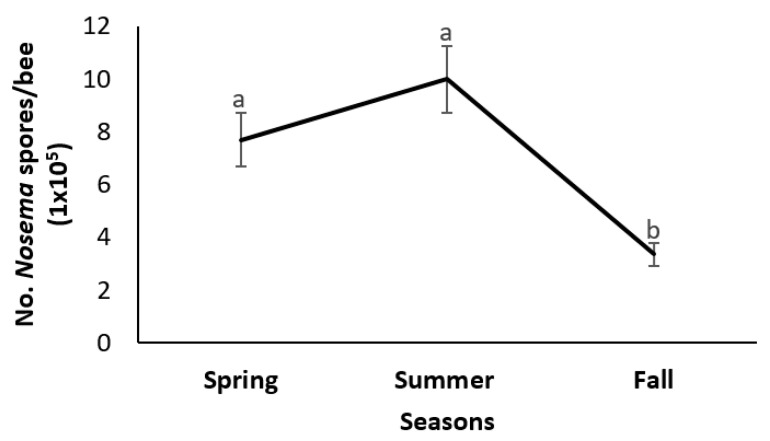
*Nosema ceranae* infection intensity (mean No. spores/bee ± SE) of honey bee colonies during spring, summer and fall (*n =* 179). Different letters indicate significant differences between seasons based on analyses of variance and Tukey tests of arcsine-square root transformed data at a significant level of <0.05. Actual data are presented.

The intensity of *N. ceranae* infections in the experimental colonies was significantly higher in the spring and summer than in the fall, and there were no differences between infection rates of spring and summer (F_2, 512_ = 30.45, *p <* 0.0001; Figure 1). Similarly, a significantly higher percentage of bees had visible *N. ceranae* spores in the spring and summer relative to the fall (F_2, 27_ = 14.04, *p <* 0.0001). Additionally, there were significant differences between seasons for the percentage of viable *N. ceranae* spores (F_2, 27_ = 63.22, *p <* 0.0001). Spore viability was approximately four-fold higher in the spring than in the summer and fall (Table 1). Furthermore, the intensity of *N. ceranae* infections was significantly correlated with the percentage of *N. ceranae* infected bees and with spore viability (r = 0.32 and 0.41, respectively, *p <* 0.01); the percentage of *N. ceranae* infected bees and spore viability were also correlated (r = 0.56, *p* < 0.0001).

### 3.2. Nosema ceranae Infections and Colony Conditions

Colonies with low levels of *N. ceranae* infection (<1,000,000 spores/bee) had significantly more bees (F_2,169_ = 3.36, *p <* 0.05), brood (F_2,154_ = 4.09, *p <* 0.05), pollen stores (F_2,148_ = 4.51, *p <* 0.05), and honey stores (F_2,154_ = 10.53, *p <* 0.0001) than colonies with high levels of *N. ceranae* infection (>2,000,000 spores/bee). They also differed with colonies having threshold levels of *N. ceranae* infection (>1,000,000 <2,000,000 spores/bee) for brood and honey stores, which were higher in the colonies with low levels of *N. ceranae* infection (Table 2). Furthermore, the intensity of *N. ceranae* infections negatively and significantly correlated with colony growth, bee populations, brood, and stores of pollen and honey (Table 3), suggesting a detrimental influence of the microsporidian on these colony variables.

The percent survivorship of overwintered colonies was lower for colonies that had high infections of *N. ceranae* during the summer (43.8%) compared to those that had threshold and low infection levels of the parasite (55.6% and 64.2%, respectively). However, these differences as well as those for colony populations and food stores were not significant (*p* > 0.05). Similarly, there was a trend in *N. ceranae* infection levels of overwintered colonies, as colonies that were classified as having high infection rates of the microsporidian in the summer had about twice the number of spores/bee in the following spring, compared to colonies that had been classified as having threshold and low infection rates of the parasite; but again, those differences were not significant (*p* > 0.05; Table 4).

### 3.3. Survivorship of Bees in Colonies

**Figure 2:**
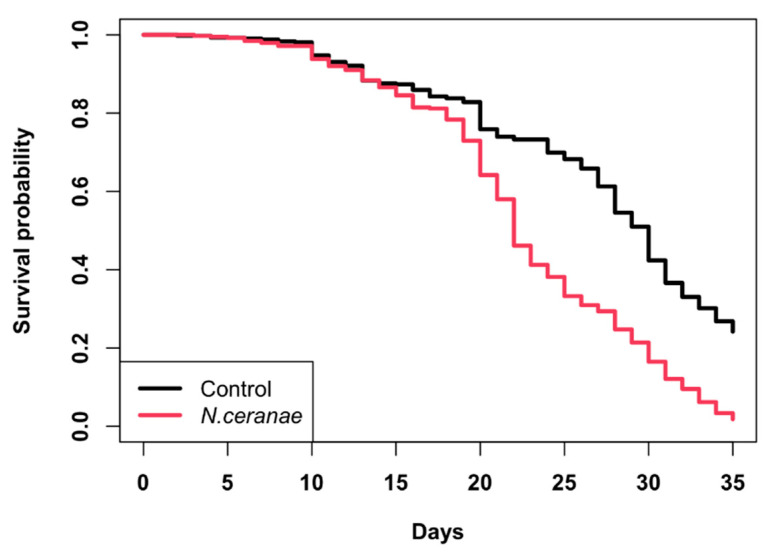
Probability of survival of adult worker honey bees inoculated with 50,000 *Nosema ceranae* spores/bee (red) or not inoculated (black) when newly emerged. Experimental bees were marked, introduced into colonies and daily censed. Survival functions were estimated with the Kaplan-Meier method.

Worker bees inoculated with *N. ceranae* spores had significantly lower survivorship rates than control bees (Long-rank test, χ^2^ = 120, *p* < 0.01; Figure 2). Additionally, at 35 days of age, only about 3% of the *N. ceranae* inoculated bees remained in the colonies vs. 25% of the control group, difference that was significant (χ^2^ = 244.6; *p* < 0.0001). The surviving bees were collected and analyzed at the end of the study, showing rates of infection of >16,000,000 spores/bee in the inoculated group of bees but <1,000,000 spores/bee in the control group. These results demonstrate that *N. ceranae* is pathogenic to honey bees in their natural environment and can impact the population growth of colonies during the seasons of the year of more bee activity.

## 4. Discussion

This study evidenced significant seasonal associations for patterns of *N. ceranae* viability, infection prevalence, and infection intensity in Canadian honey bee colonies. The study also showed that the intensity of *N. ceranae* infections is negatively related to honey bee populations, food stores and bee survivorship.

Seasonal patterns of *N. ceranae* infections were evident. Spore counts of the parasite were significantly higher in the spring and summer than in the fall. Similarly, the percentage of *N. ceranae* infected bees was also lowest in the fall, but did not differ between spring and summer. Additionally, the highest rate of *N. ceranae* spore viability was found in the spring, while the lowest rates were observed in the summer and fall. Furthermore, spore counts and rates of spore viability and infected bees were positively and significantly correlated. Contrary to these results, Jack et al. [55] found a non-significant correlation between prevalence and intensity of *N. ceranae* infections within colonies (r = 0.29, *p* > 0.05). The lack of significance of the correlation between *N. ceranae* prevalence and infection intensity in the study by Jack et al. [55] could have been due, at least partially, to the use of composite samples of bees of different ages collected from different locations in the experimental colonies, when in their study, *N. ceranae* prevalence and infection intensity differed significantly between age cohorts and sample locations. In this study, the data used for the correlation analyses were obtained from samples that in all cases were collected from the hive entrance (old bees). A previous study using bees collected from the same location in hives, also found a significant correlation between intensity and prevalence of *N. ceranae* infections [43]. Therefore, it is important to standardize methods as to the location of the hive where samples of bees are collected for diagnosis and research purposes, to make results of different studies comparable.

The epidemiological results of this study indicate that spring and summer are the most favorable seasons for the spread and development of *N. ceranae* infections. After the summer, conditions for the transmission and multiplication of *N. ceranae* decrease, as evidenced by the low rates of infection intensity, prevalence, and, above all, spore viability found in the fall. Conversely, lack of seasonality for *N. ceranae* infections has been reported from Europe and Hawaii [12,22,32], but the pattern of infection intensity and prevalence found in this study was similar to that historically known for *N. apis*, with a peak starting in mid-spring and declining in mid-summer and fall [56]. Similar results have been recently found for *N. ceranae* in Eastern and Western Canada [14,42] and in other parts of North America [13,34].

The seasonal pattern of *N. ceranae* viability, prevalence, and infection intensity is likely influenced by several factors, which include the dynamics of honey bee populations within colonies during different seasons of the year in temperate and cold climates. During late winter and early spring, the queen increases her egg-laying rate, which progressively results in higher numbers of adult bees emerging in a colony. Consequently, an increasing number of newly emerged bees rapidly acquire spores of the microsporidian by horizontal transmission via trophalaxis with older bees that overwintered and are a source of spores for new bees [57,58], or by cleaning comb cells soiled with feces contaminated with spores of the fungus [4]. This rapid transmission of microsporidian spores causes infection levels of nosema disease to spike in the spring, which remain high until the summer, because the queen continues to maintain a high egg-laying rate until the middle of that season, and many young bees continue to emerge and become infected.

While patterns of infection intensity and prevalence have been previously reported for *N. ceranae*, to the best of our knowledge, this is the first study reporting seasonal viability patterns for *N. ceranae* spores. Despite the limited knowledge on viability rates of *N. ceranae* spores in different seasons, the results of this study show that its seasonal variability coincides with the reproductive cycle of honey bee colonies. Therefore, these results suggest that the viability of *N. ceranae* spores is a relevant factor for the establishment, development, and spread of the disease. It is possible that *N. ceranae* has evolved mechanisms that allow the parasite to have high viability and infectivity rates during the seasons of maximum bee reproduction (spring and summer), which allow the microsporidian to multiply and spread more efficiently. However, despite the synchronous patterns of *N. ceranae* infections, prevalence, and spore viability, many questions remain to be answered about the fundamental causes as to why these aspects of *N. ceranae* infections fluctuate through the year; therefore, further studies are warranted. Regardless of the need for additional research, the results of this study have implications for good beekeeping practices and support the notion that the best time to apply treatments for nosema disease control is during early spring, when infection levels of the parasite rapidly grow. Future studies should also be conducted to determine if a fall treatment would be beneficial in reducing winter and spring infections of *N. ceranae*.

The negative association and correlations between the intensity of *N. ceranae* infections and colony conditions found in this study suggest that at or above threshold levels of infection (>1,000,000 spores/bee), the parasite may have a negative impact on bee populations and food stores. In fact, the colonies with threshold and high infection levels grew at a significantly slower rate than the colonies with low rates of infection. Conversely, a former study conducted in Atlantic Canada, did not find an association between *N. ceranae* infections and variation in bee populations and food stores [59]. The study by Williams et al. [59] measured colony conditions in September and only evaluated every other frame in the colonies. Perhaps if the evaluations had been conducted earlier in the summer and all frames had been assessed, the results would have been similar to ours. Additionally, our study was conducted with at least three times more colonies and the colonies were categorized according to *N. ceranae* infection levels. Higher numbers of colonies analyzed by infection levels could have increased the probability of detecting significant differences for colony conditions, particularly for colonies with high *N. ceranae* infection intensity. Similar to our findings, other studies have reported that *N. ceranae* infections may have deleterious associations with bee populations and honey yields [23,60,61], but this is the first study to show an association between degrees of *N. ceranae* infection and colony conditions. However, this, as well as previous reports, is a correlational study that reflects the association between *N. ceranae* infections and colony conditions during a snapshot in time. Therefore, studies that more precisely demonstrate cause and effect at the colony level for longer periods of time are warranted. Additionally, further research is required to more precisely determine treatment thresholds for *N. ceranae* infections during different seasons in North America. There is no consensus on the economic injury level or treatment thresholds for nosema disease caused by *N. ceranae*. Therefore, research is required to better understand injury levels for *N. ceranae* in different regions, with different bee genotypes and with different *N. ceranae* haplotypes, because all these factors can differentially and synergistically result in high or low virulence of *N. ceranae* infections in honey bee colonies. These studies would generate information to establish treatment thresholds for nosema disease that are specific for different regions. Our data support the notion that treatment is justified when infections of *N. ceranae* exceed 1,000,000 spores/bee in the region where the study was conducted.

The length of life of honey bees strongly influences colony growth, population size, and honey yields in honey bee colonies [50,52,62]. Therefore, if *N. ceranae* shortens the lifespan of bees, it could have serious detrimental effects on colony fitness and productivity. Here, we provide evidence that the survivorship rate and lifespan of honey bees in colonies are significantly reduced when workers are challenged with an inoculum of 50,000 *N. ceranae* spores/bee. The reason why *N. ceranae* infections may be associated to reduced bee populations and colony growth, as found in this study, could be explained, at least partially, by the significantly lower survival rate of the infected bees. Other studies have previously shown that *N. ceranae* infections shorten the length of life of infected honey bees. For example, Williams et al. [18], using an inoculum of 30,000 *N. ceranae* spores/bee, found that 95% of the bees in their experimental group died during a 28 to 30-day incubation period, compared to only 25% of the control group. Furthermore, Eiri et al. [63] reported that the longevity of adult bees that were experimentally infected during their larval stage with 40,000 *N. ceranae* spores/bee was reduced by 28% compared to bees that did not receive spores. Similarly, Higes et al. [16] reported that the mortality rate of bees infected with an inoculum of 125,000 spores/bee was 94.1% compared to 5.9% of the bees in the control group. The above studies, however, were conducted in laboratory settings using cage experiments. Ours is the first study to show reduced survivorship in *N. ceranae*-infected bees within the more natural environment of colonies for a period of more than 30 days, which agrees with another study conducted in hives, that showed shorter lifespans for *N. apis*-infected bees [64]. The reduction in longevity of bees infected with *N. ceranae* could be due to multiple deleterious effects of the parasite on bee health. For example, *N. ceranae*-infected workers are energetically deprived, exhibit precocious foraging, and show a decrease in the amount of vitellogenin, a protein that affects longevity in insects [11,19,65,66]. In addition, *N. ceranae* causes irreparable damage to the bees’ digestive tract, with epithelial cells of the midgut suffering lysis [67], which reduces the absorption of nutrients, leading to the premature death of the bees, as a consequence of digestive failure and starvation.

High rates of colony losses are experienced during late winter and early spring in temperate and cold climates [68,69,70]. However, *N. ceranae* infections in this study were not associated with overwintering colony conditions and mortality. Overall, the data shows a trend of more intense infections of the parasite with lower populations and lower survival rates of colonies, but this trend was not significant. It is possible that the parasite may have some influence on the survival rate and populations of honey bee colonies during winter, but apparently it is not a major cause of winter mortality as previously reported from other North American and European studies [8,10,30,31]. Conversely, this study and several others have shown that *Nosema* spp. infections detrimentally affect colony population growth during spring [23,30,60]. Therefore, this study supports the notion of low *N. ceranae* pathogenicity and virulence during winter, and high *N. ceranae* pathogenicity and virulence during spring and summer.

Based on the seasonal patterns of *N. ceranae* infection, on its association with poor colony growth, small populations, and reduced food stores during spring and summer, it would be advisable to implement control measures against the microsporidian during early spring. IPM management practices, including chemical treatments, hive sterilizations, selective breeding, and supplemental feeding, are options to manage and control nosema disease in honey bee colonies [37].

This study generated additional evidence about the epidemiology and potential negative impact of *N. ceranae* on honey bee colonies in North America, which could have detrimental consequences on colony productivity and on the pollination services that honey bees provide. The study also opens questions that warrant further investigation aimed at better understanding the impact of *N. ceranae* infections on honey bee health and productivity, and at establishing seasonal control strategies against the microsporidian.

## 5. Conclusions

This study provides data on seasonal infection intensity, prevalence, and viability of *N. ceranae* and on the relationship of *N. ceranae* infections with honey bee colony populations, food stores, bee survivorship, and overwinter colony mortality in a North American region. The highest rates of *N. ceranae* infection, prevalence, and spore viability were found in the spring and summer, while the lowest were recorded in the fall. *N. ceranae* infection levels of >1,000,000 spores/bee were significantly associated with reduced bee populations and food stores in colonies. Furthermore, worker bee survivorship was significantly reduced by *N. ceranae* infections, although no association between *N. ceranae* and winter colony mortality was found. It is concluded that *N. ceranae* infections are highest in spring and summer and may be detrimental to honey bee populations and colony productivity. Our results support the notion that treatment is justified when infections of *N. ceranae* exceed 1,000,000 spores/bee.

## Figures and Tables

**Table 1:** Prevalence and viability of *Nosema ceranae* spores in honey bee colonies during spring, summer and fall (*n =* 30).

Season	% Infected Bees ± SE	% Spore Viability ± SE
Spring	73.9 ± 1.8 ^a^	59.0 ± 6.0 ^a^
Summer	68.0 ± 1.8 ^a^	13.7 ± 1.6 ^b^
Fall	48.0 ± 5.6 ^b^	16.3 ± 1.8 ^b^

Different letters indicate significant differences between seasons based on analyses of variance and Tukey tests of arcsine-square root transformed data at a significance level of <0.05. Actual data are presented.

**Table 2:** Population and food store parameters (mean ± SE) of honey bee colonies categorized by intensity of *Nosema ceranae* infection (low, threshold, high) in the summer (*n =* 172).

Infection Category	No. Spores Per Bee ^1^	No. Frames with Bees	No. Frames with Brood	No. Frames with Pollen	No. Frames with Honey
Low	<1.0	19.7 ± 1.0 ^a^	8.4 ± 0.3 ^a^	2.1 ± 0.1 ^a^	14.1 ± 1.2 ^a^
Threshold	1.0–2.0	17.9 ± 1.3 ^a,b^	6.9 ± 0.6 ^b^	1.7 ± 0.2 ^a,b^	9.0 ± 1.3 ^b^
High	>2.0	14.3 ± 2.6 ^b^	6.7 ± 1.4 ^b^	1.4 ± 0.3 ^b^	4.6 ± 1.6 ^c^

Different letters indicate significant differences between infection categories based on analyses of variance and Tukey tests of square root transformed data at a significant level of <0.05. Actual data are presented. ^1^ millions.

**Table 3:** Correlations between infection intensity of *Nosema ceranae* (No. spores/bee), colony growth, populations, and stored food of honey bee colonies (*n =* 151).

Variables Correlated	Correlation	*p*
No. spores–Frames w/bees	−0.33	<0.0001
No. spores–Frames w/brood	−0.31	<0.0001
No. spores–Frames w/pollen	−0.24	0.0024
No. spores–Frames w/honey	−0.52	<0.0001
No. spores–Colony growth	−0.18	<0.05
Frames w/bees–Frames w/brood	0.56	<0.0001
Frames w/bees–Frames w/pollen	0.30	0.0002
Frames w/bees–Frames w/honey	0.74	<0.0001
Frames w/bees–Colony growth	0.96	<0.0001
Frames w/brood–Frames w/pollen	0.02	0.7678
Frames w/brood–Frames w/honey	0.47	<0.0001
Frames w/brood–Colony growth	0.38	<0.0001
Frames w/pollen–Frames w/honey	0.44	<0.0001
Frames w/pollen–Colony growth	0.23	<0.01
Frames w/honey–Colony growth	0.79	<0.01

**Table 4:** Populations, food store parameters (mean ± SE), and percent survival of honey bee colonies categorized by the intensity of *Nosema ceranae* infections (low, threshold, high) in the summer, that were alive the following spring (*n =* 104).

Variables	Low	Threshold	High
No. spores/bee ^1^	1.88 ± 0.25	1.75 ± 0.61	3.21 ± 1.58
No. Frames w/bees	6.3 ± 0.5	6.3 ± 0.9	5.5 ± 1.3
No. Frames w/brood	3.4 ± 0.3	4.1 ± 0.6	3.0 ± 0.5
No. Frames w/pollen	1.9 ± 0.2	2.1 ± 0.2	1.9 ± 0.9
No. Frames w/honey	8.3 ± 0.4	8.2 ± 0.7	9.7 ± 0.7
% Colony survival	64.2	55.6	43.8

^1^ millions.
